# Two giant retroperitoneal schwannomas mimicking adrenal malignancy – a case report

**DOI:** 10.1515/iss-2020-0008

**Published:** 2020-08-31

**Authors:** Isabelle Fülber, Katharina Peer, Elisabeth Maurer, Detlef K. Bartsch, Jannis Görlach, Joachim Nils Göbel, Marion Roeßler, Katharina Holzer

**Affiliations:** Department of General- and Visceral Surgery, University Hospital of Giessen and Marburg Campus Marburg, Marburg, Hessen, Germany; Department of Diagnostic and Interventional Radiology, University Hospital of Giessen and Marburg Campus Marburg, Marburg, Hessen, Germany; Department of Gastroenterology, Endocrinology, Metabolism and Infectiology, University Hospital of Giessen and Marburg Campus Marburg, Marburg, Hessen, Germany; Department of Pathology, University Hospital of Giessen and Marburg Campus Marburg, Marburg, Hessen, Germany

**Keywords:** case report, diagnosis, incidentaloma, multivisceral resection, retroperitoneal tumor, retroperitoneal schwannoma, schwannoma

## Abstract

Schwannomas are benign tumors in 95% of cases and very rarely occur in the retroperitoneum. We report the cases of a 35-year-old man with abdominal discomfort and a 50-year-old asymptomatic woman with large retroperitoneal masses. Both underwent multivisceral surgery to exclude an adrenal carcinoma, and the pathologic diagnosis showed schwannomas in both cases. Despite morphological imaging, it was not possible to get a clear diagnosis preoperatively.

## Introduction

Schwannomas are usually benign tumors and encountered in patients aged 20–50 years and in women twice as often as men. Schwannomas mostly arise from Schwann cells of cranial and peripheral nerves in the head, neck or upper extremities. They extremely rarely occur in the retroperitoneum (only 1–3% of all schwannomas) where they grow very large before diagnosis and consequentially cause complications due to their size. So, retroperitoneal schwannomas have a greater tendency to undergo spontaneous degeneration and hemorrhage compared to other locations [1].

Diagnosis is based on histopathologic examination and immunohistochemistry. The identification of Antoni A and Antoni B regions is the histologic hallmark of schwannomas. Antoni A areas appear more organized and are composed of compact spindle cells arranged in short bundles or interlacing fascicles, whereas Antoni B regions are hypocellular and loosely organized. They contain more myxoid and edematous tissue. Lesions are mostly encapsulated and S-100 protein positive in immunohistochemically analysis [2].

Due to the lack of sensitivity to radiation and chemotherapy, surgical resection is the first-line treatment, and an adjuvant therapy is not recommended. Since schwannomas are mostly benign tumors, prognosis after removal is very good with a very low risk of recurrence. A follow-up care is recommended after removal of benign retroperitoneal schwannomas [3].

## Case report

### Case 1: 35-year-old male

A 35-year-old male was admitted to our university hospital for further examination by his general practitioner (GP). The patient presented with abdominal pain and diarrhea. A large intraabdominal mass (13 × 12 cm) was detected via ultrasound by the GP. Because of his body mass index (33.2 kg/m^2^), the mass was not detected on physical examination. An abdominal computed tomography scan (CT) showed a mass of 12 × 11 × 12 cm in the left upper retroperitoneum likely originating from the left adrenal gland. The mass showed signs of calcification, as well as cystic areas, toward the center. Multiple, small retroperitoneal lymph nodes were seen with a maximum size of 15 × 7 mm. Besides the repressive growth of the mass, the surrounding structures showed no signs of pathology. A thoracic CT scan showed no signs of metastases. As far as is known, family history of this patient was negative for tumors or syndromes.

**Figure 1:**
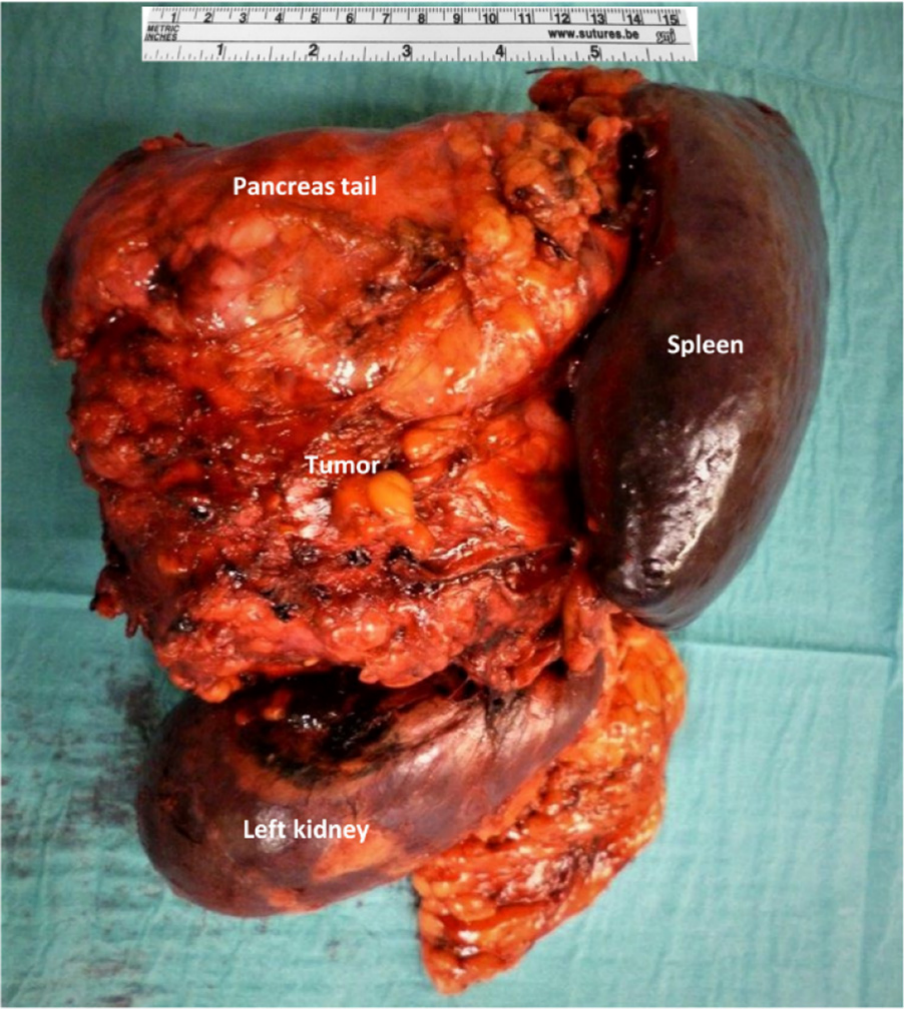
Surgical specimen of the male patient. Surgical specimen of the male patient showing a strong adherent pancreas tail, spleen and left kidney with the later defined schwannoma.

With the suspected diagnosis of a suprarenal tumor, like pheochromocytoma, cortical adenoma or adrenal carcinoma, an endocrine diagnostic test was performed. There was no sign of hormonal activity of the tumor as the aldosterone-to-renin ratio was normal, the dexamethasone suppression test was negative, adrenal steroids were unremarkable and plasma and urinary metanephrine and normetanephrine were within the normal range. For differential diagnosis, an interferon-gamma release assay, as well as serology for echinococcosis, was performed but also negative. All other blood parameters, besides slightly elevated inflammation values, were within normal ranges. The blood pressure, heart rate and oxygenation were normal. With the differential diagnosis of an adrenal carcinoma or neuroblastoma, the interdisciplinary tumor conference recommended removal of the large tumor (Figure 1).

**Figure 2:**
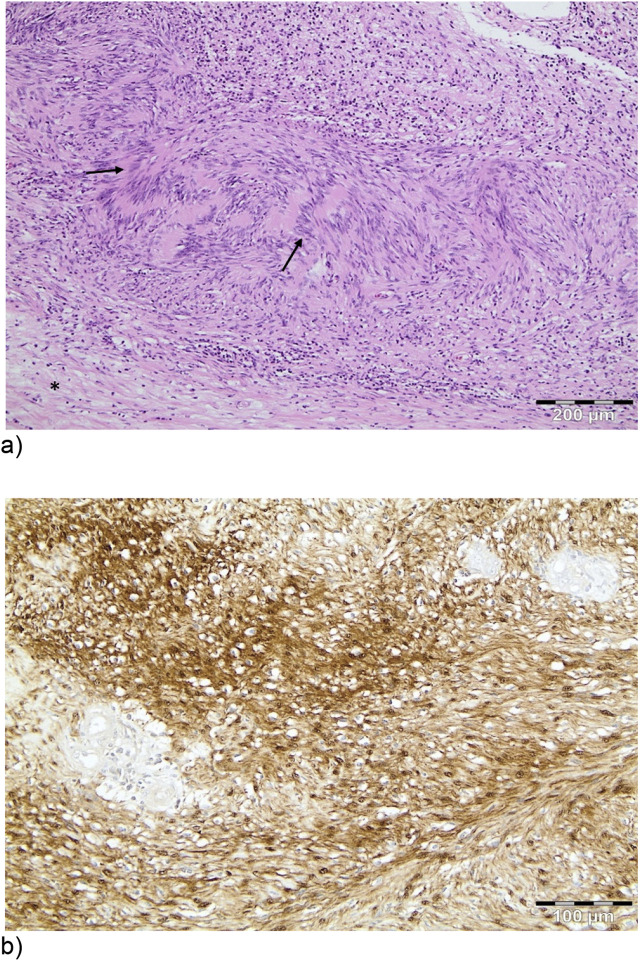
Histopathological findings. (a) 11.5 cm spindle cell tumor with cellular compact Antoni A areas (arrows), alternating with hypocellular Antoni B areas (*). Antoni A areas are composed of interlacing bundles of spindle cells (Schwann cells) occasionally discrete aggregates termed Verocay bodies (H&E, ×100). (b) Immunnoprofile: positive stain for S-100 protein (×200).

**Figure 3:**
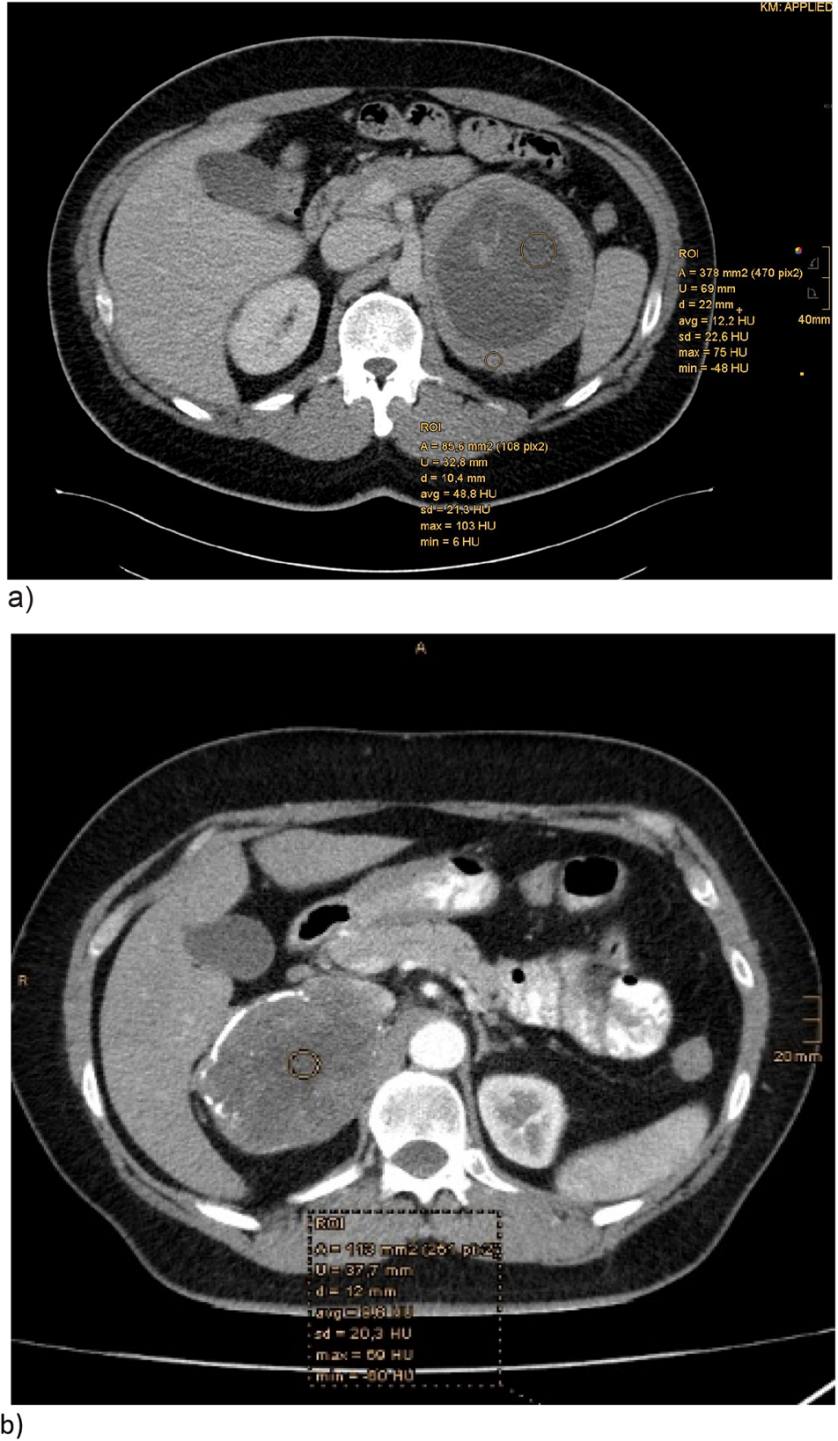
Contrast-enhanced CT scans of both patients. (a) Male patient with a mass of the left adrenal gland. Central parts have 12 HU on average and peripheral areas have about 49 HU in average. Cystic areas, as well as septa, can be seen in this section, central calcifications on further images. (b) Female patient with adrenal tumor of the right adrenal gland displacing the vena cava inferior and an infiltration cannot be barred. Also cystic elements and calcifications are visibly. The tumor has about 9.6 HU on average in the central parts.

Intraoperatively, a cystic and solid mass was found in the left retroperitoneum with adhesions to the left kidney, the splenic vessels, the pancreatic tail and the tumor enforced the left suprarenal gland. Broad adhesions of the tumor to the surrounding structures, as well as strong adhesions, of the splenic vein required splenectomy, nephrectomy and partial left sided pancreatectomy in order to completely resect the tumor (Figure 3a).

In pathology, the tumor was 11.5 × 10.2 × 9.5 cm and surrounding the left adrenal gland. Macroscopically, it had solid and cystic areas as described in the CT. The cystic areas were most likely to derive from circulatory disturbances within the tumor. Microscopically, a schwannoma was diagnosed. Immunohistochemically, staining revealed positivity for S-100, vimentin and negativity for CK MNF 116, Melan A and inhibin (Figure 2). The Ki67 proliferation index was 5–8%. No tumor mass or infiltration could be detected in the pancreas, the kidney or the spleen. All 24 removed lymph nodes (paraaortal, subdiaphragmatic/paraesophageal, peripancreatic and omental) were free of tumor cells.

Postoperative care included the adaption to the single kidney with a transient polyuria and postsplenectomy management. The patient was discharged in a good general condition and will be continuing regular medical aftercare.

First aftercare was done three months after surgery with an abdominal magnetic resonance imaging (MRI), clinical examination as well as checking renal function and function of the pancreas. In all these checkups, no dysfunction was detected. The MRI showed no local relapse.

### Case 2: 58-year-old female

In the course of a routine checkup with ultrasound, her GP found an asymptomatic adrenal mass in the right retroperitoneum. In further clarification through an abdominal CT, this lesion showed a size of 9.2 × 6.2 cm displacing the inferior vena cava to the left. The patient was sent to our department of endocrinology for further examinations.

Also, in this patient, a full endocrine workup showed no hormonal activity of the tumor suspected to be of adrenal origin. Levels of catecholamine in serum and urine, as well as of renin, aldosterone, cortisol, testosterone, were in the normal range. Because the external radiological report did list a pheochromocytoma as one of the main differential diagnosis and the patient suffered of occasional headaches and cardiac palpitations, a metaiodobenzylguanidine-(MIBG) scintigraphy (adrenal scan) was additionally performed. The MIBG scintigraphy showed no increased tracer accumulation regarding chromaffin cells. A thoracic CT scan showed no signs of metastases, just an unspecific solitary pulmonary nodule. With the suspected diagnosis of an adrenal carcinoma, the tumor with strong adhesions to surrounding structures was resected (Figure 3b). Therefore, a resection of the right adrenal gland and tangential resection of the inferior vena cava and the right kidney with the ureter were necessary. Histopathological examination also showed a schwannoma in this case. The tumor was capsulated, spindle shaped with a diameter of 8 cm. Despite strong adhesions, intraoperatively, no invasion to surrounding structures was determined. Immunohistochemically, staining revealed positivity for S-100, partial positivity for glial fibrillary acidic protein and negativity for CK MNF 116 and synaptophysin. The Ki67 proliferation index was less than 1%. Also in this case, all resected paraaortal and paracaval lymph nodes and lymph nodes of the right renal artery, as well as of the hepatoduodenal ligament, were free of tumor cells. This patient was discharged in a good general condition as well and will be continuing regular medical aftercare.

## Discussion

Expecting an adrenal carcinoma, we removed two giant retroperitoneal tumors with strong adhesions to surrounding structures. Intraoperatively, we performed a multivisceral resection. Surprisingly, histopathological findings defined the tumors in both cases as benign retroperitoneal schwannomas.

Just one percent of all retroperitoneal neoplasms are schwannomas [1]. They have a low rate of recurrence and very good prognosis. They nearly never undergo secondary malignant transformation. Most of them were incidental findings during clarification of another disease or a routine checkup. In most cases, they are round and encapsulated with a diameter of about several centimeters with displacing growth. The main treatment of peripheral schwannomas is surgical resection. Because of their displacing and noninfiltrative growth, a multivisceral resection is not indicated. In case of malignant schwannomas additional to surgical resection, chemotherapy is an opportunity [4].

Because retroperitoneal schwannomas are quite rare and lack specific symptoms, a correct preoperative diagnosis is often missed. Typical misdiagnoses include pancreatic cysts, liver tumors, psoas abscesses or, as in our cases, tumors of the adrenal gland. Preoperative exact establishment of diagnosis is especially challenging in cases of retroperitoneal schwannomas that adhere to other structures [3]. Once an adrenal mass is detected, it is mandatory to determine whether the lesion is malignant or benign and whether it is hormonally active or nonfunctioning. Schwannomas do not show hormonal activity.

Differential diagnoses of hormonally inactive adrenal tumors include nonfunctioning adrenal adenomas, adrenal carcinomas, metastases or mesenchymal or stromal tumors (for example, myelolipoma or schwannomas). Despite numerous investigations, it is often not possible to exclude malignancy preoperatively in tumors finally classified as benign schwannomas.

The best available data regarding the classification of an adrenal mass as benign exist for noncontrast CT scans, in which a homogenous tumor with Hounsfield units ≤10 is consistent with a benign lesion. The risk of an adrenal incidentaloma harboring a primary adrenal carcinoma varies from 4 to 25%, depending on the size of the tumor, so that the size of an incidentaloma can be additionally used to predict malignancy. Furthermore, an age <40 years, as in case 1, makes the diagnosis of an adrenal carcinoma more probable [5], [6].

Typical features of CT images of schwannomas include smooth and sharp margins with an enhanced appearance, as well as liquefaction, necrosis and hemorrhage within the tumor; these features are nonspecific. But because of these features, schwannomas can be differentiated from lymphomas, hemangiomas, cysts and connective tissue diseases in most cases [2], [3], [7], [8]. On CT scans, adrenal carcinomas may show peripheral enhancement with central nonenhanced areas of necrosis, calcifications or distant enlarged lymph nodes. Characteristic features of malignancy like irregular margins, lack of capsule, local invasion and distant metastases are not always present. Adrenocortical carcinomas have a poor prognosis, and the only chance of cure is the complete surgical resection [5].

In both of our cases, the imaging showed large oval tumors of the adrenal gland with a well-circumscribed and mild contrast-enhanced margin. Both tumors were more than 9 cm in diameter. The tumors were not homogenous as they contained internal septa and central calcifications. Hounsfield units of the central parts of the tumor were around 12 (case 1, venous phase) and around 9.6 (case 2, early arterial phase), but peripheral areas Hounsfield units were high (49 HU). There were no signs of enlarged lymph nodes or distant metastases. However, in both cases, it was difficult to differentiate whether the tumors were only displacing or infiltrating the neighboring tissue.

Fine-needle aspiration biopsy (FNA) of a suspected adrenal mass is generally not recommended by the European Society of Endocrinology (ESE) and European Network of adrenal tumor (ENSAT) guideline unless there is a history of extraadrenal malignancy [6]. There exist two small retrospective case series on endoscopic ultrasound-guided FNA (EUS-FNA) of retroperitoneal schwannomas: In the first series, all three tumors (with a size of max. 27.4 mm) could be successfully diagnosed as schwannomas. In the second series, only four out of six, with a maximum diameter of 40 mm, were correctly classified [9], [10]. So, EUS-FNA could be helpful in cases of suspected smaller, asymptomatic retroperitoneal schwannomas as with a definite diagnosis of a benign schwannoma observation might be sufficient.

Because of large size, heterogeneous appearance, calcifications and possible infiltration of surrounding structures, both tumors could not be classified in preoperative imaging as benign [6]. In addition, large retroperitoneal schwannomas consist often of a heterogeneous appearance and multiple regressive proportions, so FNA can be misleading. Due to this, frozen section examination is also heterogeneous and difficult to define dignity. It would not be possible to exclude malignancy confidently in both our cases. In our two cases, infiltrative growth and malignancy was suspected, and so, primary resection was performed in accordance with the guideline.

Maybe in case of smaller schwannomas with lesser broad adhesions to surrounding organs, it might be possible to clarify the entity in frozen section examination so that a frozen section should be performed to prevent patients of multivisceral resection.

In our two cases, infiltrative growth and malignancy was suspected, and so, primary resection was performed in accordance with the guideline.
