# Supplementary material for: Two giant retroperitoneal schwannomas mimicking adrenal malignancy – a case report
Source: Innov Surg Sci. 2020 Aug 31;5(1-2):75–9. doi: 10.1515/iss-2020-0008 (PMC7798304; doi:10.1515/iss-2020-0008)
Supplement: Supplementary file 1 [file iss-5-20200008-s001.pdf]

Reviewer Assessment

Isabelle Fülber 3t al.: Two giant retroperitoneal schwannomas mimicking adrenal malignancy – a case report

Reviewers’ Comments to Original Submission

Reviewer 1: anonymous

Date received: 12-May-2020
Reviewer recommendation: **Return to author for minor modifications**
Reviewer overall scoring: **High**

Assessment Form scores: 5 = High/Yes; 3 = Medium/Adequate; 1 = Low

Is the subject area appropriate for the journal					
Does the title clearly reflect the paper's content?	5				
Does the abstract clearly reflect the paper's content	5				
Do the keywords clearly reflect the paper's content?				2	
Does the introduction present the problem clearly?	5				
Are the results/ conclusions justified?			3		
How comprehensive and up-to-date is the subject matter presented?	5				
How adequate is the data presentation?	5				
Are units and terminology used correctly?	5				
Is the number of cases adequate?	5				
Are the experimental methods/ clinical studies adequate?					
Is the length appropriate in relation to the content?	5				
Does the reader get new insights from the article?		4			
Please rate the practical significance.		4			
Please rate the accuracy of methods.			3		
Please rate the statistical evaluation and quality control.			3		
Please rate the appropriateness of the figures and tables.					
Please rate the appropriateness of the references.	5				
Please evaluate the writing style and use of language.	5				
Please judge the overall scientific quality of the manuscript.		3			
Are the methods used worthy of reproduction in greater deal?	No				

Comments to author: The current study presents two rare and interesting cases of very large retroperitoneal neurinomas. Retroperitoneal tumors can reach large sizes before symptoms occur.The majority (80%) of primary retroperitoneal tumors are either primarily malignant or have a tendency to secondary malignancy.The surgical procedure described by the authors

corresponds to the current guidelines of the Surgical Working Group Endocrinology of the German Society for General and Visceral Surgery (DGAV) (2017) for tumors larger than 6 cm. Since neurinomas almost never undergo secondary malignant transformation and do not grow infiltratively, tumour resection including removal of neighbouring organs is not indicated. The authors should please point out the possibilities of a preoperative diagnostic confirmation or intraoperative frozen section examination and the conclusions drawn therefrom. In particular, the resulting change in therapeutic procedure.

Reviewer 2: anonymous

Date received: 12-Jun-2020
Reviewer recommendation: **Return to author for minor modifications**
Reviewer overall scoring: **High**

Assessment Form scores: 5 = High/Yes; 3 = Medium/Adequate; 1 = Low

Is the subject area appropriate for the journal					
Does the title clearly reflect the paper's content?	5				
Does the abstract clearly reflect the paper's content	5				
Do the keywords clearly reflect the paper's content?					1
Does the introduction present the problem clearly?		4			
Are the results/ conclusions justified?	5				
How comprehensive and up-to-date is the subject matter presented?		4			
How adequate is the data presentation?		4			
Are units and terminology used correctly?		4			
Is the number of cases adequate?		4			
Are the experimental methods/ clinical studies adequate?	5				
Is the length appropriate in relation to the content?		4			
Does the reader get new insights from the article?			3		
Please rate the practical significance.		4			
Please rate the accuracy of methods.		4			
Please rate the statistical evaluation and quality control.		4			
Please rate the appropriateness of the figures and tables.		4			
Please rate the appropriateness of the references.		4			
Please evaluate the writing style and use of language.		4			
Please judge the overall scientific quality of the manuscript.		4			
Are the methods used worthy of reproduction in greater deal?	No				

Comments to author: Well-presented cases, however, the Keywords do not reflect the Content of the paper. I suggest the following Keywords: retroperitoneal Tumor - schwannoma - diagnosis - multivisceral resection

Authors' Response to Reviewer Comments

Date received: 22-06-2020

Response to reviewer 1

Dear Reviewer 1,

I would like to extend my appreciation to your comment to our case report. I confirm that we acted in accordance with the principle of ESE and ENSAT guideline. I just pointed out, that in case of an encapsulated schwannoma with non-infiltrative growth a multivisceral resection is not indicated (see changes below).

If the tumor is huge, it is often difficult to even find the origin of the tumor, which can lead to misdiagnosis. In both of our cases preoperative diagnosis showed a retroperitoneal tumor with the suspected origin of the adrenal gland. An infiltrative growth was suspected after computed tomography so that we needed to exclude malignancy. Because of its size both tumors showed regressive changes in about 80% of the tumor mass and just 20% showed specific tissue so it might have been a lucky coincidence to get a diagnosis in frozen section examination. But to support your comment, a frozen section examination is a well available opportunity for intraoperative diagnosis. So I corrected this mistake (see changes below) and completed the diagnostic capabilities.

Changes: p.5, discussion: Just one percent of all retroperitoneal neoplasms are schwannomas (1). They have a low rate of recurrence and very good prognosis. They nearly never undergo secondary malignant transformation. Most of them were incidental findings during clarification of another disease or a routine check. In most cases they are round and encapsulated with a diameter of about several centimetres with displacing growth. The main treatment of peripheral schwannomas is surgical resection. Because of their displacing and non-infiltrative growth, a multivisceral resection is not indicated. The only exception is in case of malignant schwannomas where in addition to surgical resection chemotherapy is an opportunity (4).

p. 7, discussion: Because of the large size, the heterogeneous appearance, the calcifications, the possible infiltration of surrounding structures both tumors could not be classified in preoperative diagnostic as benign structures (6). Because symptoms often appear at a very late stage retroperitoneal schwannomas are often very large. This mostly causes a high proportion of severe regressive variations and a heterogeneous appearance. Due to this the appearance in frozen section examination is also heterogeneous and difficult to define dignity or diagnosis based on a small tissue sample. Even with an intraoperative frozen section examination it would not be possible to exclude malignancy confidently in our both cases so resection in anticipation of an adrenal carcinoma was performed.

In case of smaller tumors with less regressive changes it might be possible to clarify the entity in frozen section examination so that a frozen section examination should be performed to potentially prevent patients of multivisceral resection.

Response to reviewer 2

Dear Reviewer 2,

Thank you a lot for your review and your short comment. I think you're right with the point of the keywords, there was a mistake. I changed them and also added the keywords "multivisceral resection" as well as "diagnosis".

Comments by the Editor-in-Chief to Revised Submission

All reviewer concerns raised were addressed satisfactorily. The manuscript may be published in the present stage.
